# Time Course-Dependent Study on Equine Herpes Virus 9-Induced Abortion in Syrian Hamsters

**DOI:** 10.3390/ani10081369

**Published:** 2020-08-07

**Authors:** Osama Abas, Walied Abdo, Samy Kasem, Abdulatif Alwazzan, Asmaa G. Saleh, Ibrahim G. Saleh, Hideto Fukushi, Tokuma Yanai, Mohie Haridy

**Affiliations:** 1 Department of Pathogenetic Veterinary Sciences, United Graduate School of Veterinary Sciences, Gifu University, Gifu 501-1193, Japan; dr_osamaabas2020@yahoo.com (O.A.); hfukushi@gifu-u.ac.jp (H.F.); 2 Department of Animal Medicine, Faculty of Veterinary Medicine, Alexandria University, 21526 Alexandria, Egypt; 3 Department of Pathology, Faculty of Veterinary Medicine, Kafrelsheikh University, 33516 Kafrelsheikh, Egypt; waliedsobhy@yahoo.com; 4 Department of Virology, Faculty of Veterinary Medicine, Kafrelsheikh University, 33516 Kafrelsheikh, Egypt; samykasem2@gmail.com; 5 Department of Veterinary Diagnostic Laboratories, Ministry of Environment, Water and Agriculture, Riyadh 11195, Saudi Arabia; a.alwazzan83@gmail.com; 6 Department of Animal Medicine, Faculty of Veterinary Medicine, Damanhur University, 22511 Damanhur, Egypt; dr_asmaaghalep@yahoo.com; 7 Department of Pharmacology and Toxicology, Faculty of Pharmacy, Al-Azhar University, 11651 Cairo, Egypt; iisaleh44@gmail.com; 8 Laboratory of Wildlife and Forensic Pathology, Biomedical Science Examination and Research Center, Department of Veterinary Medicine, Faculty of Veterinary Medicine, Okayama University of Science, Imabari, Ehime 794-8555, Japan; 9 Department of Pathology and Clinical Pathology, Faculty of Veterinary Medicine, South Valley University, 83523 Qena, Egypt

**Keywords:** EHV9, hamster, early and late trimester, cytokines, abortion

## Abstract

**Simple Summary:**

Equine herpesvirus 9 (EHV-9) is a virus belonging to the family of equine herpesviruses. EHV-9 has been isolated from natural infections of different wild and zoo animals. In addition, it has been associated with encephalitis and abortion in several animal species. However, the host range and pathogenesis of this virus are still unknown. Herein, we investigated the underlying pathogenesis of EHV-9-induced abortion in relation to the gestation period in either early or late trimester infection. We noticed that the late trimester infection of EHV-9 was associated with more severe death and both placental and fetal tissue localization of the virus. Also, early stage infection was accompanied by band necrotic changes within the placenta, which usually led to abortion.

**Abstract:**

This study aimed to follow the time-course pathogenesis of EHV-9 abortion in early and late trimesters. Twenty-seven pregnant hamster dams were divided into three groups: (G1) control, (G2) EHV-9-inoculated on the 5th day (early trimester), and (G3) EHV-9-inoculated on the 10th day of gestation (late trimester). Dams were sacrificed at different time points during gestation and examined for viremia and viral DNA in different fetal and maternal tissues and pathological changes in fetal tissue, placenta, and cytokines. Animals in G3 showed a marked increase in the number of dead fetuses than those in G2. Histopathological findings of G2 showed early band coagulative necrosis of maternal spaces and stromal decidual cells. Necrotic changes were observed within the decidua basalis, spongiotrophoblast layer, and labyrinth. First, the virus was localized within mononuclear leukocytes in the decidua capsularis and basalis, and within the necrotic chorionic villi and cervical epithelium. G3 demonstrated degenerative changes within the chorionic villi and trophospongium. The virus antigen was observed within the chorionic villi, trophoblasts, mononuclear cells, and fetal tissues. In conclusion, EHV-9 induced abortion mostly occurs through necrosis of the chorionic villi and cannot cross through the capsular placenta in the early trimester but can through the developed decidual placentation.

## 1. Introduction

Equine herpesvirus 9 (EHV-9) is the newest member of the family of equine herpesviruses belonging to the subfamily *Alphaherpesvirinae* genus *Varicellovirus*. The virus was isolated from Thomson’s gazelles (*Gazella Thomsoni*) that died in a Japanese zoo from fulminated encephalitis. Previously, it was called gazelle herpesvirus 1 (GHV-1) but later was renamed EHV-9 due to its close genetic homology to other members of the equine herpesvirus family [1,2]. The homology between EHV-9 and equine herpesviruses 1 and 8 (EHV-1, EHV-8) was estimated to be approximately 95%, while it was 60% to equine herpesvirus 4 (EHV-4), based on analysis of the glycoprotein G and the conserved region of glycoprotein B gene sequences [1]. At the same time, EHV-9 is distinguishable from EHV-1 by DNA fingerprinting analysis [1]. EHV-9 can infect various domestic animals and nonhuman primates, causing fatal infections with fulminate encephalitis, which is characterized by neuronal degeneration and necrosis as well as intranuclear inclusion bodies in rodents [1,3,4,5], marmosets [6], dogs [7], cats [8], goats [9], pigs [10,11] and cattle [12]. Intranasal inoculation was the most common method of virus administration in the experimental studies; a strong affinity of EHV-9 for the olfactory system as the site of viral replication has been observed in all animals experimentally expressing encephalitis [11,13].

Although EHV-9 has been isolated from natural infections of Thomson’s gazelles (*Gazella Thomsoni*) [1], a giraffe [14,15], a polar bear, Gravy’s zebras, and a Persian onager [16,17], the natural host reservoir and the complete host range of EHV-9 are wide, although they are still mostly unknown [17,18,19].

Previously, the pathological findings of EHV-9 abortion were investigated in mice, hamsters [4] and rats [5] induced in the early and late trimesters. This study aims to follow up the time-course pathogenesis of EHV-9 abortion in both the early and late trimesters to provide detailed descriptive data on the pathogenesis of abortion during EHV-9 infection by monitoring a variety of parameters such as detection of viremia, viral DNA in different fetal and maternal tissues, and pathological changes in fetal tissue, as well as associations with the placenta and cytokines to abortion at different time points.

## 2. Materials and Methods

### 2.1. Virus

EHV-9 propagation was performed using Madine Derby bovine kidney (MDBK) cells. The inocula used for infection were prepared by culturing the virus from original seed stocks of EHV-9 (P19, 5th passage in MDBK cells) in MDBK cells. Then, the virus was titrated by a plaque forming assay on MDBK cells to detect the titer.

### 2.2. Animals and Treatments

Twenty-seven 10-week-old pregnant female Syrian hamsters (*Mesocricetus auratus*) were purchased from a breeder (SLC Inc., Hamamatsu, Japan) on the third day of gestation to be used for EHV-9 inoculation in the early, or first, trimester. Six animals were purchased from the same breeder on the eighth day of gestation to be used for EHV-9 inoculation during the end of the late, or third, trimester. Both groups were acclimatized for 2 days before virus inoculation. The animals were housed in an isolated biohazard cabinet, fed basal pellets (Oriental MF, Oriental Yeast Co., Tokyo, Japan) and given bottled sterilized water ad libitum. The guidelines and ethics of animal experimentation were approved by the Animal Care and Use Committee of Gifu University (approval ID: EA16-20). The appropriate Institutional Animal Care Guidelines were followed during all handling and procedures. The animals were inoculated intranasally with 50 µL (1 × 104 pfu) of EHV-9 virus solution divided between both nasal openings. The virus was inoculated, under deep anesthesia, on the fifth day of gestation in the early trimester group and on the tenth day of gestation in the late trimester group.

Three hamster dams in group 2 were sacrificed at the third, sixth, and eleventh days post-inoculation (pi) (full term). Three control dams (group 1) were sacrificed at the same time points. Group 3 animals were sacrificed at the third and fifth days post-infection of EHV-9. Similarly, dams in group 1 were sacrificed at the same time points. The experimental design is illustrated in Table 1. The animals were observed daily for any clinical signs or evidence for abortion, and animals were sacrificed by overdose of inhalation anesthesia, and careful postmortem examination was performed immediately for sample collection.

### 2.3. Collection and Processing of Samples

Heparinized blood samples were collected for the detection of viral DNA in the blood. Raw blood samples were collected for serum separation for the detection of cytokines. 

### 2.4. Histopathology and Immunohistochemistry

Samples of the dam’s brain, lungs, liver, spleen, uterus, and placenta and whole fetuses were collected and fixed in 10% neutral buffered formalin. After fixation, the tissues were dehydrated and embedded in paraffin wax, sectioned (5 mm), stained with hematoxylin and eosin (HE), and examined by light microscopy. Sections of the placenta, uterus, brain, lungs, spleen, liver of the dams, and whole fetuses, including the brain, lungs, and livers were immunolabeled with rabbit polyclonal serum specific for EHV-9 (1:800) by using the two-step polymer method (EnVision+ system HRP labeled polymer anti-rabbit, Dako, Tokyo, Japan) according to the manufacturer’s instructions. Positive and negative control tissues were used in the set of staining. The EHV-9 antiserum was produced in the Laboratory of Veterinary Microbiology, Gifu University. The sections were counterstained with hematoxylin after immunostaining.

### 2.5. Enzyme-Linked Immunosorbent Assay for Detection of Cytokines (IFN-γ and TNF-α) 

The levels of two important cytokines, interferon-gamma (IFN-γ) and tumor necrotizing factors (TNF-α), were measured in the serum samples of EHV-9 inoculated pregnant hamsters at different time points and compared to the control counterparts to investigate their roles in the induction of abortion. Hamster TNF-α (TNF superfamily, member 2) and Hamster IFN-γ ELISA Kits (Biosource Inc., San Diego, CA, USA) were used. Samples were measured in duplicate and standard [20]. The absorbance was measured by an I Mark Microplate Absorbance Reader (Bio-Rad, Hercules, CA, USA) in dual-wavelength measurement mode with a 450 nm filter and 630 nm as a reference filter for IFN-γ and *TNF-α*, respectively, as recommended in the kit instructions. The concentration of the samples was obtained through analysis of the standard curve using Microsoft Excel 2010.

### 2.6. DNA Extraction and Polymerase Chain Reaction

Tissues (50 mg) from the maternal brain, lungs, liver, spleen, uterus, placenta, and fetuses (brain, lung, and liver tissues) were collected for viral DNA extraction. Samples were collected in Eppendorf tubes on ice and homogenized using a sterilized plastic tissue homogenizer and suspended in 500 µL PBS. DNA was extracted using a SepaGene nucleic acid extraction kit (Sanko Junyaku Co., Tokyo, Japan) according to the manufacturer’s instructions, and samples were kept at −80 °C until PCR was performed. 

A total of 111 samples of maternal tissues (84) and fetal tissues (27) were tested by conventional PCR for EHV-9 viral DNA. Two primers, Orf30 sense 5′-GTC AGG CCC ACA AAC TTG AT-3′ and Orf30 antisense 5′-ATA GGA GTC TGT GCC GTT GT-3′ targeting DNA polymerase (Orf30) of EHV-9, were designed using the Snap gene 2.6 software to be used in conventional PCR for amplification of 214 base pairs. The PCR amplification was performed in 25 mL volumes containing DNA 2 µL, 2 µL dNTPs, 1 µL (10 µMol µM) of each primer, 2.5 µL 10× Ex. Tag buffer, 0.25 µL Ex. Tag polymerase (Takara, Kyoto, Japan), and 17.25 µL distilled water. The PCR conditions were as follows: 5 min at 94 °C (initial denaturation), 35 cycles of 5 s at 98 °C, 30 s at 47 °C, and 45 s at 72 °C; and 7 min at 72 °C (final extension). The PCR products were separated on an agarose gel (1.5%) and stained with ethidium bromide.

### 2.7. Statistical Analysis

Statistical analysis was performed by one-way analysis of variance followed by the Tukey Cramer test for multiple comparisons *p* < 0.05 using Graph Pad prism software version 5 (San Diego, CA, USA).

## 3. Results

### 3.1. Clinical Signs and Gross Findings

#### 3.1.1. Inoculation of EHV-9 in First Trimester Hamsters

**Figure 1:**
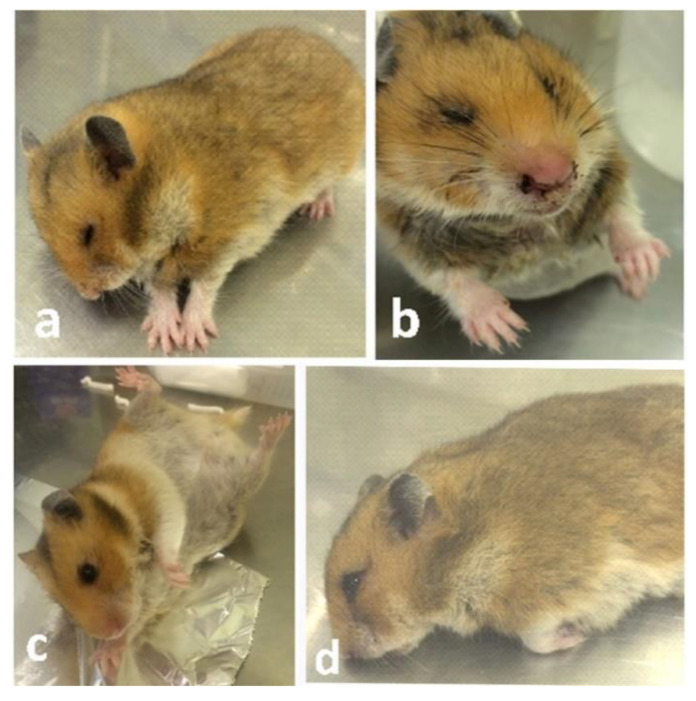
(**a**) Pregnant hamster inoculated with EHV-9 at the 1st trimester (5th day of gestation) suffered from head tilting and paddling at 3 dpi. (**b**) Pregnant hamster inoculated with EHV-9 at the 1st trimester (5th day of gestation) suffered bilateral nasal bleeding at full term (11th dpi). (**c**) Pregnant hamster inoculated with EHV-9 at the last trimester (10th day of gestation) exhibited convulsions at 6 dpi. (**d**) Pregnant hamster inoculated with EHV-9 at the last trimester (10th day of gestation) exhibited paralysis of hindquarter and recumbency at the 6th dpi.

The neurological clinical signs appeared on the third day post-inoculation (dpi) and became severe at the fifth dpi and onwards until the end of the experiment. Some cases showed recumbency and paralysis at the end stage. Dams inoculated with EHV-9 in the early trimester (fifth day of gestation) rapidly progressed to neurological signs consisting of tremor and convulsions with paddling as well as nasal discharge and ruffled fur at the third dpi (Figure 1a). At the sixth dpi, the neuronal manifestations were severe convulsions and sometimes spasms in the whole body with increased nasal discharge. At full term of pregnancy (11th dpi), most of the animals showed severe convulsions with lateral recumbence and severe dyspnea. One animal showed bilateral blindness with nasal bleeding (Figure 1b).

**Figure 2:**
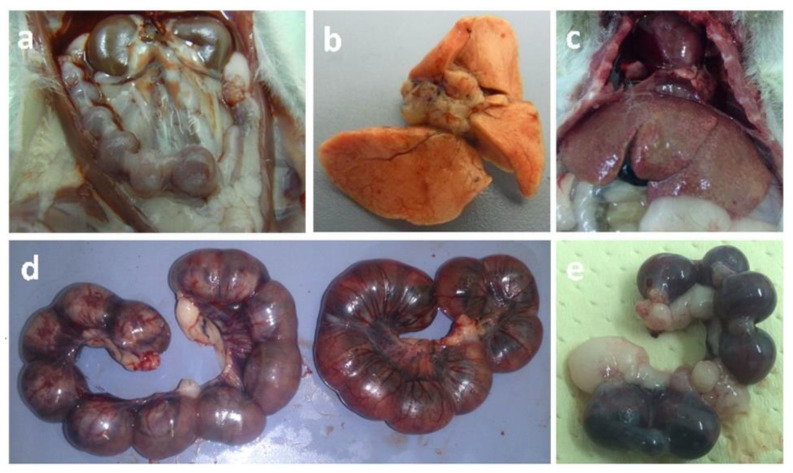
(**a**) The uterus of pregnant hamster inoculated with EHV-9 at the 1st trimester (5th day of gestation) revealed an ischemic white pregnancy cyst in comparison with surrounding cysts at 3 dpi. (**b**) Liver of pregnant hamster inoculated with EHV-9 in the 1st trimester (5th day of gestation) revealed pinpoint necrosis of the liver with sharp demarcation of lobules, 6 dpi. (**c**) Lungs of pregnant hamster inoculated with EHV-9 in the 1st trimester (5th day of gestation) revealed pinpoint hemorrhages, 3 dpi. (**d**) The uterus of pregnant hamster inoculated with EHV-9 in the 1st trimester (5th day of gestation) revealed severe congestion with hemorrhagic dark areas (right) at 6 dpi compared to control gravid uterus (11 days of gestation). (**e**) The uterus of pregnant hamster inoculated with EHV-9 in the 1st trimester (5th day of gestation) revealed a small dark pregnancy cyst in comparison with surrounding cysts at 6 dpi.

The most prominent gross pathological finding at the third dpi was congestion of the pregnant uterus. One dam revealed a small transparent empty vesicle of the pregnancy cyst (Figure 2a). Petechial hemorrhages and pinpoint necrotic foci were observed in the lungs (Figure 2b). At the sixth dpi and at full term, dams exhibited splenomegaly and hepatomegaly with pinpoint hepatic necrosis (Figure 2c). Moreover, meningeal congestion in the olfactory area, petechial to patchy hemorrhage, congestion of pulmonary tissue, and ascites with the accumulation of gelatinous fluid (1 dam) were noted. The uteri were severely congested, blood-tinged, and blackish discolored in some cases (Figure 2d). The small undersized transparent uterine vesicles with absence of motility were evident for fetal death at 6 dpi (Figure 2e). At full term, undersized dark congested dead fetuses were collected and compared to fresh alive ones.

#### 3.1.2. Inoculation of EHV-9 in Late-Trimester Hamsters

**Figure 3:**
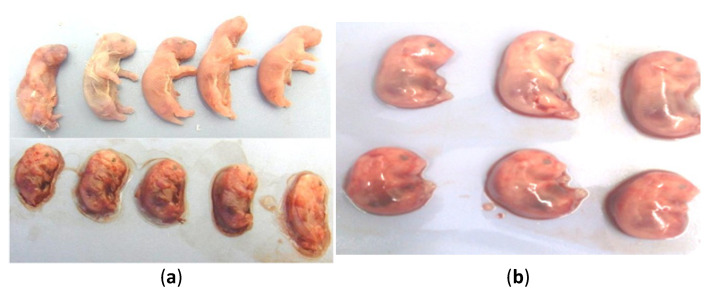
(**a**) Pups of pregnant hamsters inoculated with EHV-9 in the early first trimester (5th day of gestation) were congested, undersized, and found dead or stillbirth (lower row) at 11th dpi in comparison with the control (upper row) at parturition time (16 days of gestation). (**b**) Pups of pregnant hamsters inoculated with EHV-9 in the late trimester (10th day of gestation) were congested, undersized fetuses (lower raw) at 3 dpi in comparison with control fetuses at the 13th day of gestation (upper row).

The clinical signs in the late trimester inoculated group were similar to those observed in the early trimester inoculated group, but the signs progressed rapidly and appeared more prominent and severe. Convulsions, tremors, and hindquarter paralysis and recumbency (Figure 1c,d) were observed. The gross findings were congestion and sharp demarcation of hepatic lobules. Hepatic, pulmonary, meningeal, and uterine congestion were detected. Ascites and hepatomegaly were noted in only a few cases. At 3 dpi, the affected fetuses were congested and undersized in comparison with those of control dams on the 13th day of gestation (Figure 3a). The uteri were dark red and contained small dead, congested, undersized fetuses at 6 dpi in the last trimester animals. Dead fetuses accounted for 12/30 in the late trimester virus-inoculated group in comparison with 0/28 mice in the control group.

A summary of clinical signs and gross findings is provided in Table 2. The live fetuses were detected by motility and the symmetrical size of the uterine vesicles, while the dead fetuses exhibited undersized uterine vesicles with signs of inflammation with absence of motility. The number of dead fetuses in each dam and the corresponding control dams is summarized in Table 2.

### 3.2. Histopathology and Immunohistochemistry

#### 3.2.1. Infection During the First Trimester of Gestation

**Figure 4:**
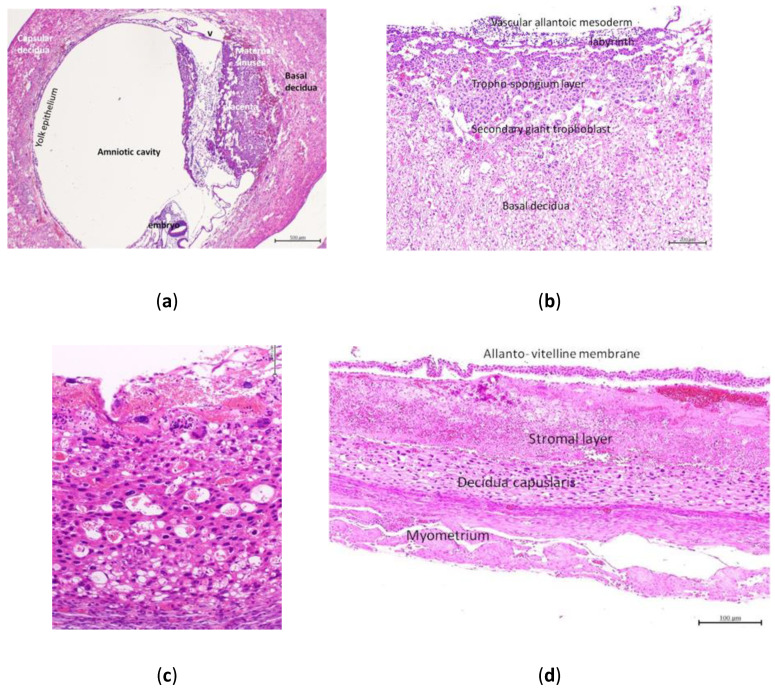
The 8th day of gestation in the control group: the placentation cone composed of the implantation site, basal decidua, and capsular decidua (**a**). The implantation site composed of vascular allantoic mesoderm, labyrinth, trophospongium layer, secondary giant trophoblasts, and basal decidua (**b**). The capsular placentation (yolk sac placentation) composed of yolk sac epithelium, secondary giant trophoblasts, and capsular decidua (**c**). Cross section through the capsular placentation and myometrium revealed allanto-vitelline membrane, stromal layer, decidua capsularis and myometrium (**d**).

On the eighth day of gestation in the control group, the placentation cone showed the vascular allantoic mesoderm membrane, a narrow labyrinth with fetal blood vessels separated from maternal blood by a thin layer of trophoblast, a zone of irregularly arranged trophoblast cells permeated by lacunae full of maternal blood, the trophospongium layer, and a layer of secondary giant trophoblast cells followed by a thick layer of the decidua basalis (Figure 4a,b). The maternal blood vessels in the decidua basalis were invaded by migratory trophoblasts and intraluminal polymorphonuclear leukocytes (PMNLs). The ante-mesodermal side revealed an inverted visceral wall of the yolk sac, yolk sac cavity, a layer of secondary giant trophoblast cells, and a thick layer of the decidua capsularis (Figure 4c). The giant trophoblast layer was permeated by numerous large lacunae full of maternal blood. The maternal blood spaces contained intraluminal PMNLs. Amorphous eosinophilic materials occupied the extensive intercellular spaces in the decidua capsularis. Few multinucleated syncytial giant cells were observed. Different layers of the capsular placenta are illustrated in Figure 4d.

**Figure 5:**
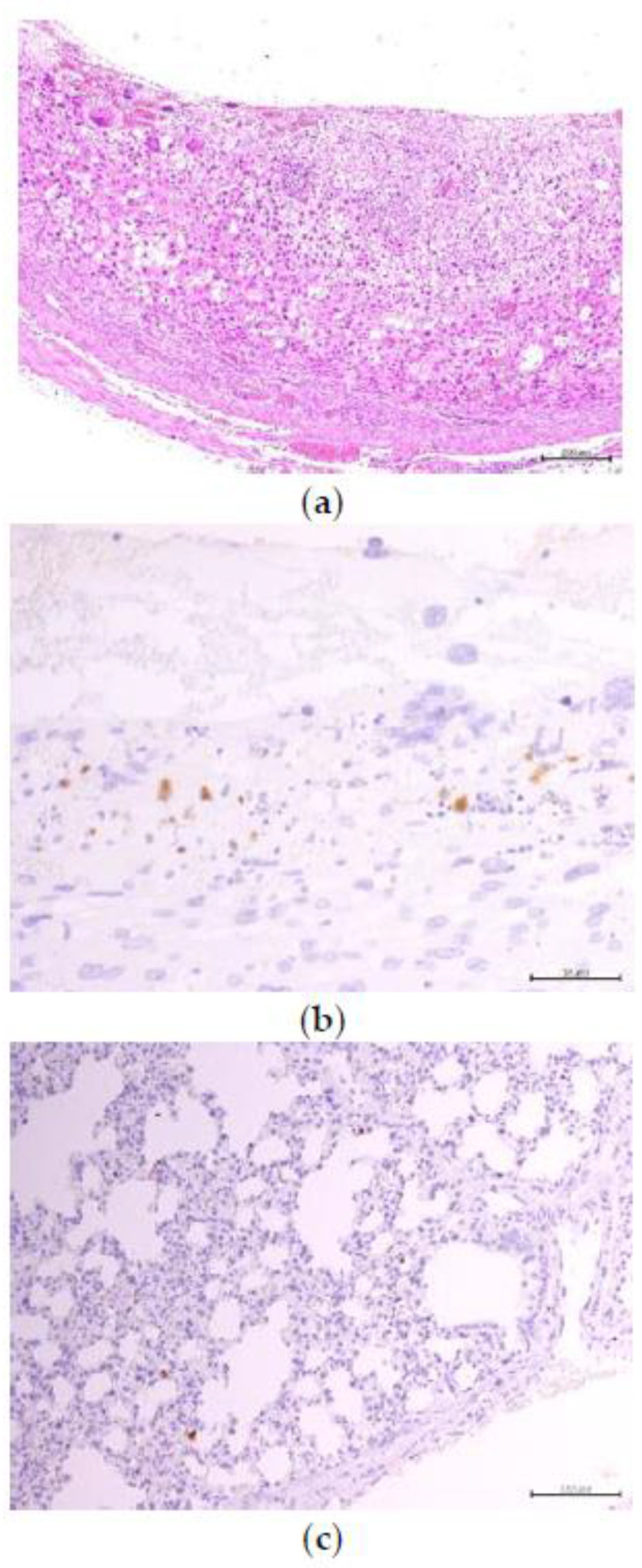
Placentas of hamsters in group 2 revealed coagulation in the maternal spaces and stromal decidual cells in the decidua capsularis (**a**). The virus antigen was detected in mononuclear leukocytes in areas of necrosis in the decidua capsularis (**b**), and a few alveolar macrophages (**c**) were stained positive in dam no. 3. IHC, polymer horseradish peroxidase technique, Mayer’s hematoxylin counterstain.

At 3 dpi in group 2, the capsular giant trophoblast cells were necrotic with pyknotic nucleus with degenerated atrophied cytoplasm and were permeated by spaces of maternal blood. Coagulative necrosis was observed in the maternal spaces and stromal decidual cells, which were more severe in the decidua capsularis. Multinucleated syncytial giant cells were observed in the necrotic area (Figure 5a). In the placentation cone, intravascular migratory trophoblasts were degenerated and attacked by leukocytes. In the case of dead embryos, the pregnancy cyst was smaller and the excleome was narrower. The embryonal tissue degenerated, the ectoplacental cone was ill-developed, and the decidua capsularis was severely necrosed with focal dystrophic calcification. The surrounding blood vessels were engorged with PMNLs and macrophages. The number of foreign body giant cells surrounding the necrotic stromal decidual cells increased.

Immunohistochemically, the virus was detected in mononuclear leukocytes in areas of necrosis in the decidua capsularis in dam no. 3. Positive mononuclear cells were observed around the giant syncytia in necrotic areas (Figure 5b). In the brain, dispersed neuronal degeneration besides vacuolation of nerve bundles and minimal lymphocytic infiltration were observed in the olfactory bulbs of the three dams. Focal meningitis was observed in dam no. 3. Multifocal mild interstitial pneumonia associated with bronchial desquamation was observed in the pulmonary tissue of dam no. 3. Immunohistochemically, few neuronal cells and mononuclear cells were stained positively in the olfactory bulbs of the three dams. A few alveolar macrophages stained positive for dam no. 3 (Figure 5c).

On the 11th day of gestation, the placental disc was composed of the labyrinth layer, including fetal capillaries, basement membrane, syncytiotrophoblasts 1 and 2, cytotrophoblasts and maternal blood spaces, trophospongium, and thin decidua basalis. At the periphery of the placental disc, there were still a few secondary giant cells; however, the decidua capsularis was very thin or completely absent at the tubal and vaginal ends of each gestation sac. The fetus started differentiation and the chorionic villi of the chorio-allantoic placenta were developed.

**Figure 6:**
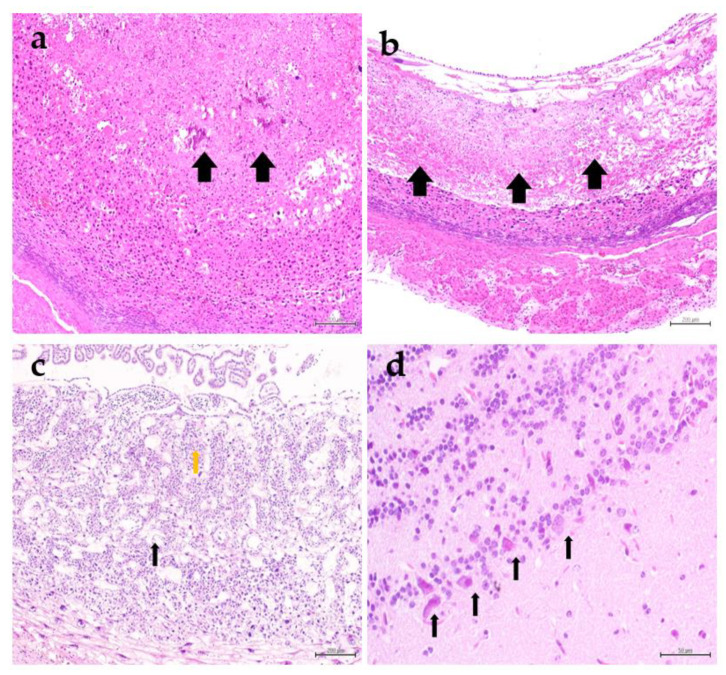
Pregnancy cysts of hamsters in group 2 at 6 dpi revealed dead fetuses that appeared as necrosed tissue (arrows) occupying the lumen (**a**). The decidua capsularis revealed liquefactive necrosis (arrows) (**b**). The placental disc revealed vacuolated decidua basalis (black arrow), vacuolation, necrosis, and hemorrhage of the spongiotrophoblast layer and labyrinth (orange arrow) (**c**). The neurons of olfactory bulb in dams were degenerated and contained intranuclear eosinophilic inclusions (**d**), H&E.

**Figure 7:**
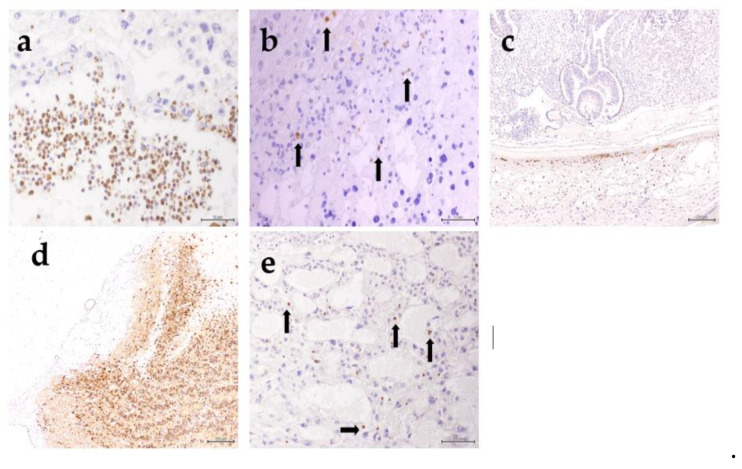
The viral antigen was detected in fetal tissue in group 2 at 6 dpi in mononuclear leukocytes occupying the maternal blood spaces in the thin decidua capsularis and basalis (**a**), degenerated trophoblast cells in the labyrinth and spongiotrophoblast layer (**b**), and leukocytes infiltrating the necrotic areas in the decidua capsularis, in contrast to the live fetal tissue (**c**). The antigen was also observed in the olfactory bulb (**d**) and lungs (**e**) of dams in group 2 at 6 dpi.

At 6 dpi, one dead fetus was observed in each dam. The pregnancy cyst of the dead fetus was involuted, and the lumen was occupied by the necrosed fetal tissue (Figure 6a). The decidua capsularis showed liquefactive necrosis (Figure 6b). The placental disc revealed vacuolated decidua basalis, vacuolation, necrosis, and hemorrhage of the spongiotrophoblast layer as well as the labyrinth with foreign body-type giant cell formation (Figure 6c). Other pregnancy cysts were similar to those of the control group, and few cytotrophoblast cells were degenerated. The neurons of the olfactory bulb in dams were degenerated and contained intranuclear eosinophilic inclusions (Figure 6d). Lymphocytic infiltration also containing nuclear inclusions was observed. The lungs, liver, and spleen of each of the three dams revealed minimal pathological changes. Immunohistochemically, the mononuclear leukocytes occupying the maternal blood spaces in the thin decidua capsularis as well as the decidua basalis were stained positive for the virus antibody (Figure 7a). Few degenerated trophoblast cells in the labyrinth and spongiotrophoblast layers were stained positive (Figure 7b). The leukocytes infiltrating the necrotic areas in the decidua capsularis were stained positively, while alive fetal tissue was negatively immunostained (Figure 7c). Positively stained leukocytes were observed in several pregnancy cysts of dam no. 5. A few leukocytes were positive in one or more pregnancy cysts in dams nos. 4 and 6. In the dead mice, few cells stained positive for maternal blood spaces. The degenerated neurons in the olfactory bulb (Figure 6d), cerebrum, and mononuclear cells were stained positive. Dispersed immunostained mononuclear cells were dispersed in the pulmonary (dams no. 4, 5) (Figure 6e) and hepatic and splenic tissues (dam no. 5).

On the 15th day of gestation, full birth fetuses had fully developed allanto-chorionic placentas composed of chorionic villi, degenerated labyrinthine, and trophospongium layers with a very thin vacuolated decidual layer.

**Figure 8:**
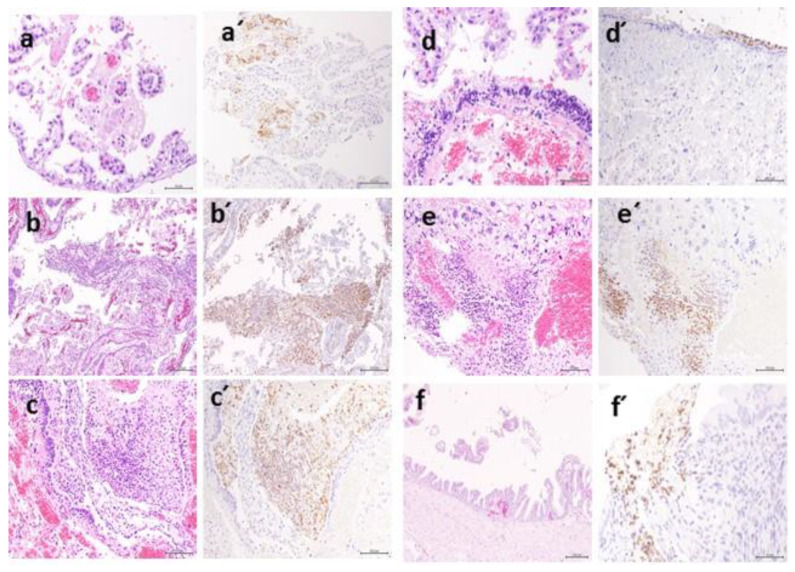
Multifocal necrosis in chorionic villi (**a**), spongiotrophoblast areas which anchor the placenta into the uterine lining (**b**,**c**), Hyalinization of the Reichert’s membrane associated with necrosis and inflammatory cell infiltration (**d**) and focal necrosis around maternal blood spaces rich with mononuclear cells (**e**) and cervical epithelium necrosis (**f**). (**a’**–**f’**): Immunohistochemistry revealed positive staining of the affected tissue referred to as dashed letters corresponding to images stained with H&E.

At 11 dpi, multifocal necrosis of chorionic villi was observed at the junction with labyrinthine associated with mononuclear inflammatory cell infiltration (Figure 8a). Necrosis was observed in the spongiotrophoblast areas, which anchor the placenta into the uterine lining (Figure 8b,c). Hyalinization of the Reichert’s membrane was associated with focal hyperplasia of the overlying endoderm with inflammatory cell infiltration (Figure 8d). Necrotic mononuclear cells around the maternal blood vessels near the junction of the uterine wall and trophospongium were observed. Focal necrosis in the areas surrounding the maternal blood spaces sometimes showed engorgement with mononuclear cells (Figure 8e). The cervical epithelium was necrotic and infiltrated by mononuclear cells, and shreds of necrotic desquamated materials clung to it (Figure 8f). None of the dead or live fetuses (5 fetuses per dam were examined) showed pathological lesions. Immunohistochemically, the focal necrotic chorionic villi were stained positive against anti-EHV antibody (Figure 8a′). Immunostaining of the chorionic epithelium was observed intra-cytoplasmically, sometimes related to chorionic stroma. The necrotic debris clung among chorionic villi stained positively to the virus antibody (Figure 8b′,c′). The necrotic material and inflammatory cells around Reichert’s membrane were positive (Figure 8d′). Necrotic areas and mononuclear cells in the lumen and around maternal blood vessels at the junction of trophospongium and uterine wall were also positive (Figure 8e′). The necrotic epithelium of the cervix reacted positively (Figure 8f′). The liver and lungs of the examined mice were not stained positively except for cellular debris swallowed in bronchioles and alveoli.

The brain revealed neuronal degeneration with intranuclear inclusion bodies in the olfactory bulb. The affected neurons stained positive for the antibody. The pulmonary tissue revealed focal areas of hemorrhages related to a diffuse mild degree of interstitial pneumonia observed in dam no. 7; however, focal areas of aggregations of mononuclear and leukocytic cells were frankly dispersed in pulmonary tissue sometimes related to blood vessels or bronchi as seen in dam no. 9. Dispersed alveolar cells stained positively for EHV. The hepatocytes were sparsely immunostained against EHV antibodies in the two dams.

#### 3.2.2. Infection During the Third Trimester of Gestation

Placentation at 13 days of gestation revealed a well-developed mature placenta composed of chorionic villi, maximum-size labyrinthine, and trophospongium layer, and a thin layer of the decidua basalis. The fetus had differentiated organs.

**Figure 9:**
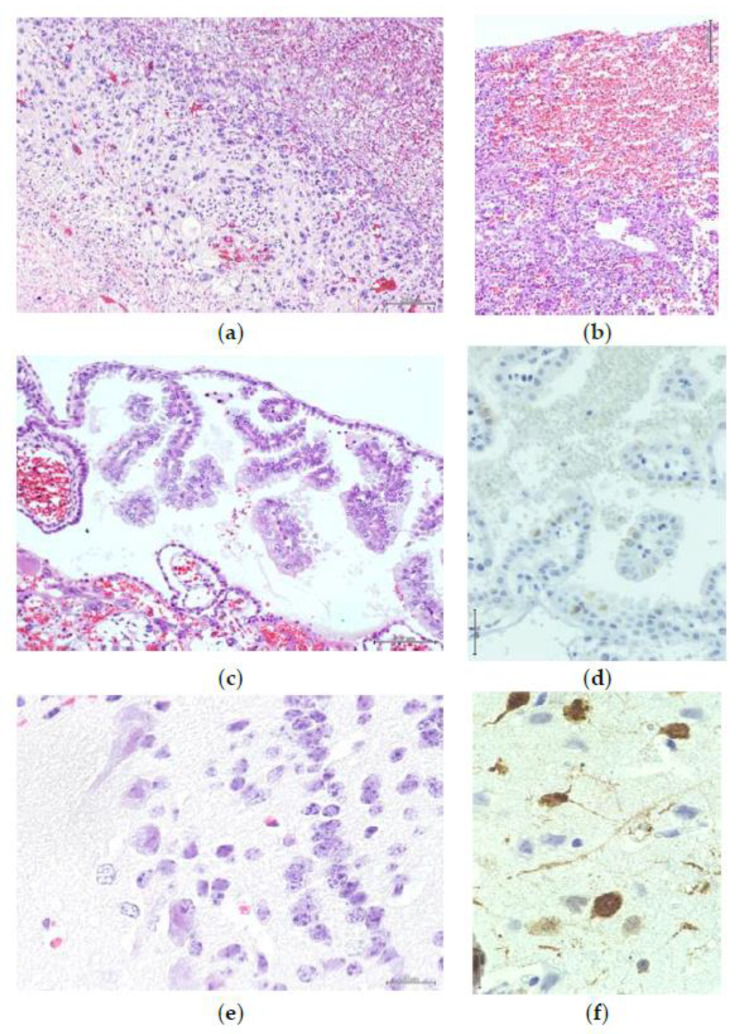
Placental lesions in hamsters of group 3 at 3 dpi revealed minute focal necrosis in the decidua basalis (**a**) associated with hemorrhages in the placental tissue and around fetuses (**b**). Chorionic epithelium revealed granular pale apices (**c**) positively immunostained for the Equine herpesvirus (EHV) antigen (**d**). The neurons of the olfactory bulb were degenerated and necrotic, containing eosinophilic intranuclear inclusions (**e**) that were positively immunostained for EHV antigen (**f**).

At 3 dpi, minimal pathological changes were observed in the uterine wall, including minute focal necrosis in the decidua basalis (Figure 9a) related to maternal blood vessels, sometimes resulting in hemorrhage circumscribing the whole fetus and separating the placenta from the uterus (Figure 9b). These changes were observed in dam no. 12 and to a milder degree in dam no. 10. The chorionic epithelium revealed granular pale apices (Figure 9c). Hemorrhage and unicellular to minute focal necrosis were observed in the feet of dam no. 12. The neurons of the olfactory bulb were degenerated and necrotic, containing eosinophilic intranuclear inclusions (Figure 9e). These lesions extended to affect the lower pyramidal neurons of the cerebral cortex in dams 10 and 12. Lesions were localized in the granular cell layer of the olfactory bulb in dam no. 11. The hepatic tissue was severely congested with central hydropic degeneration in dam no. 12. Unicellular necrosis was also observed. Mild similar changes were also observed in dam no. 10. Immunohistochemically, the antibody specifically stained the cytoplasm of the chorionic epithelium (Figure 9d) in the placentas of some fetuses in dams 10 and 12. Few cells around necrotic areas in the decidua were also immunostained. Some hepatocytes were positively stained intracytoplasmically, especially in necrotic cellular aggregations in fetuses of dam no. 12. The affected neurons in the olfactory bulb and the pyramidal layer of the cerebral cortex stained positive for the EHV antigen (Figure 9f). The EHV antigen has been sparsely detected in some hepatocytes in dam no. 12.

**Figure 10:**
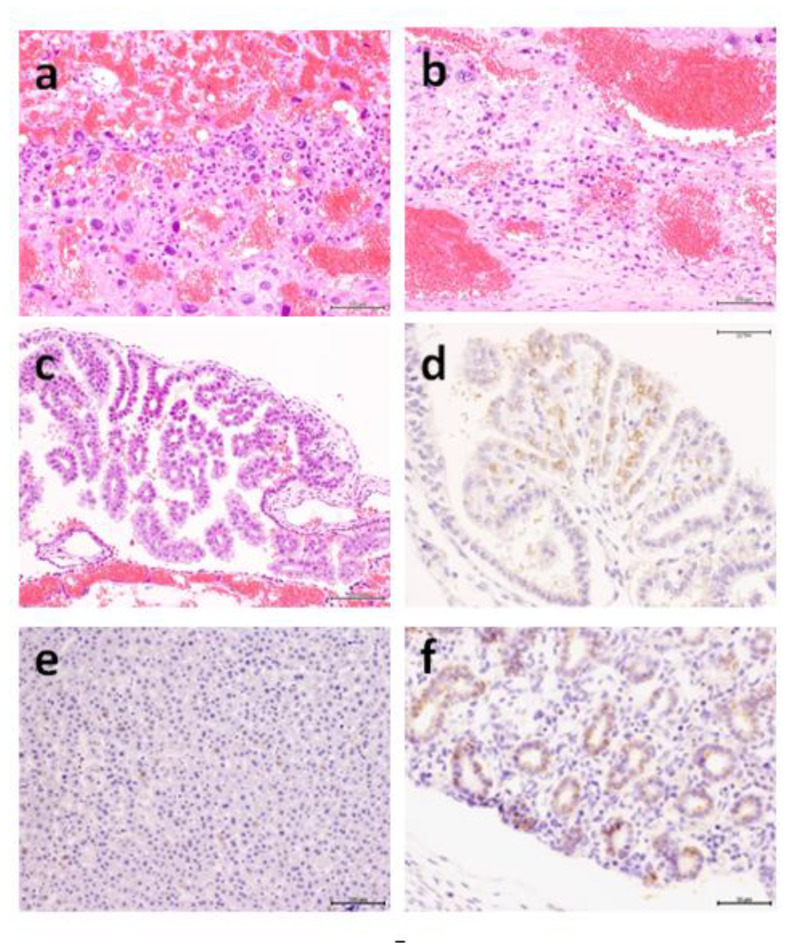
Placental lesions in hamsters in group 3 five-dpi revealed leukocytic infiltration in the trophospongium (**a**), focal necrosis in thin decidua basalis with focal hemorrhage (**b**), and degeneration and hemorrhages of chorionic villi (**c**). Virus antigen was detected in the chorionic villi (**d**) and fetal hepatic (**e**) and pulmonary tissues (**f**).

At 5 dpi, mononuclear infiltration was observed in the trophospongium layer close to the labyrinthine layer (Figure 10a). Dispersed degeneration of trophoblast cells occurred in the trophospongium and labyrinthine layers. Focal necrosis around the maternal blood spaces was observed in the thin layer of decidua basalis (Figure 10b). Mild degenerative changes and hemorrhage were observed in and between the chorionic villi (Figure 10c). Hemorrhages were observed in fetal tissues, and the uterine wall was separated from the placenta in dam no. 14. Immunohistochemically, the virus antigen was detected in the chorionic villi (Figure 10d), a few trophoblasts in the labyrinth, and in mononuclear cells around the necrotic areas in the decidua stroma. Antigens were detected in hepatic tissue (Figure 10e) and in the pulmonary epithelium of fetuses (Figure 10f). The brain was congested with microscopic hemorrhage in the neuropil in dam 13. Perivascular monocytic infiltration as well as dispersal focal neurosis of neurons occurred throughout the brain tissue in dam 13. Focal neuronal degeneration with intranuclear eosinophilic inclusions was observed in the medulla oblongata of dam no. 14. The virus antigen was detected in the olfactory bulb, part of the frontal lobe adjacent to the bulb, and focally in the pyramidal cells of the cerebral cortex. Moreover, the antigen was detected in the focal degenerated neurons in the medulla oblongata in dam 14. Vacuolation of the hepatic cell cytoplasm was observed in dam no. 14. Immunostaining in Kupffer macrophages was positive. Focal interstitial pneumonia was observed in dams 13 and 14. Diffuse interstitial pneumonia with excessive edema was observed in no. 15. Immunostaining of alveolar macrophages and pulmonary epithelium was sparsely detected in dams no. 13 and 14.

### 3.3. Detection of Viral DNA by PCR

EHV-9 DNA was amplified in blood samples (viremia) from the third to the sixth dpi, but it could not be detected at other points of time. Viral DNA was detected in the brain tissues of dams from the third dpi until the full term of gestation in both the early and late trimester infection groups. In uterine tissue, the viral DNA was detected starting from the sixth dpi until full term in early trimester infection and at the third dpi until full term in the late trimester infection group.

The virus could not be detected or isolated from placental and fetal tissues at the third and sixth dpi in group 2, so PCR detection was not applicable. Viral DNA was detected in placental tissue at full birth and at the third dpi until full birth in groups 2 and 3, respectively. In fetal tissues (brain, lung, and liver), viral DNA was not detected in groups 2 and 3, except for two fetal liver and lung samples at full term in group 3, as illustrated in Table 3.

### 3.4. Detection of Cytokines (IFN-γ and TNF-α) by ELISA Kits

**Figure 11:**
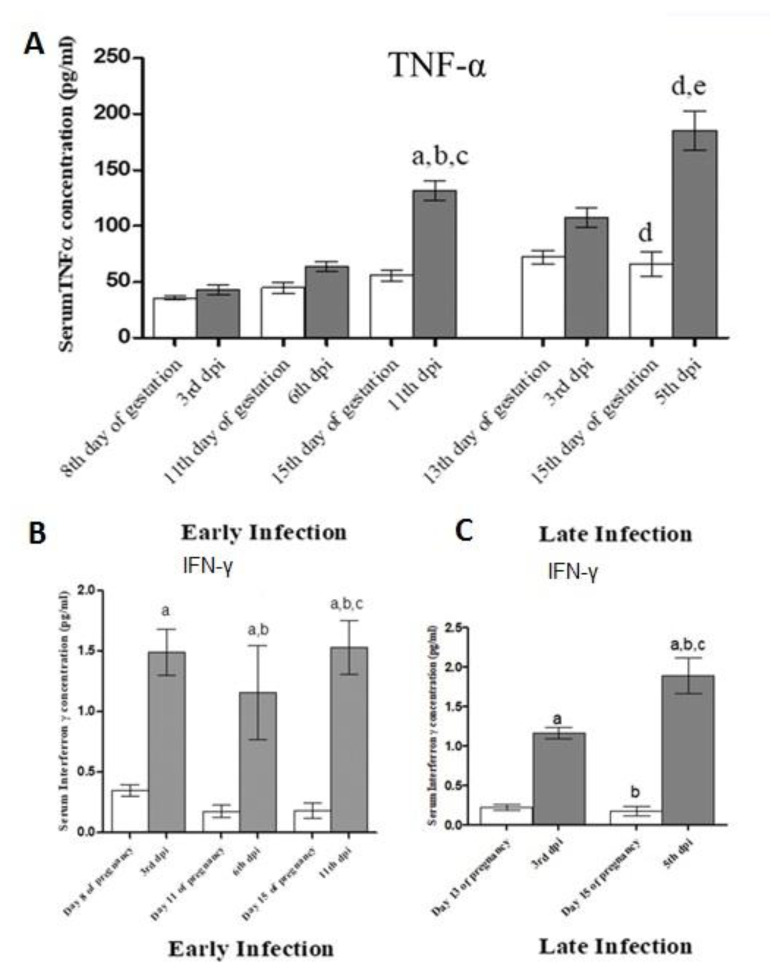
(**A**) Serum concentrations of TNF-α in hamsters inoculated with EHV-9 (1 × 10^4^ pfu) in early and late trimesters in comparison with the control group. (**B**) and (**C**) Serum concentrations of IFN-γ in hamsters inoculated with EHV-9 (1 × 10^4^ pfu) in early (a) and late (b) trimesters in comparison with the control group. Data are represented as mean ± SE. Statistical analysis performed by one-way analysis of variance followed by the Tukey Cramer test for multiple comparisons (*p* < 0.05). The letters (a–e) superscripted the columns are referred to the significance between groups represented in the graphs.

There were gradual increases in the levels of TNF-α at the 3rd, 6th, and 11th DPI and the 3rd and 6th DPI of EHV-9 in groups 2 and 3, respectively. The levels of TNF-α were significantly higher at the 11th and 5th DPI in groups 2 and 3, respectively, than those of the control group (Figure 11A). The level of IFN-γ significantly increased at the 3rd, 6th, and 11th DPI and the 3rd and 6th DPI of EHV-9 in groups 2 and 3, respectively, when compared with those of the control non-inoculated group The levels of IFN-γ significantly and linearly increased throughout the time points of the experiment in both groups 2 and 3.

Serum TNF-α levels were significantly increased at the 11th DPI of group 2 when compared to those at the 3rd and 6th DPI. In addition, they significantly increased at the 6th DPI when compared to those at the 3rd DPI in group 3. Serum IFN-γ levels were significantly increased at 11th DPI in group 2 when compared to those in the 3rd and 6th DPI Serum IFN-γ levels were significantly increased at the 6th DPI in group 3 when compared to those in the 3rd DPI (Figure 11B,C).

## 4. Discussion

Encephalitis has been induced by EHV-9 in various animal species [7,8,9]; however, the pathogenicity of EHV-9 infection in pregnant animals has not yet been elucidated. Infection with EHV-9 during gestation may potentially impact the breeding of many domestic animals, as it is known that the virus has a wide host range [18]. This study investigated the time-course effects of EHV-9 infection in pregnant hamsters in the early and late trimesters to follow up the pathogenesis of abortion induced by EHV-9. EHV-9 has a close immunological relationship with EHV-1, which was estimated to be 95% [1]. The significance of EHV-1 in the horse-breeding industry is important as it is a common cause of respiratory disease, abortion, neonatal death, and, more rarely, neurological signs in this species [21]. Although EHV-9 has been isolated from natural wild animal infections with nervous and abortive disorders [16,17], its significant role in disease outbreaks has not yet been explained [19]. EHV-9-induced abortion has been experimentally investigated in mice, rats, and hamsters [4,5] when the placenta was fully developed. In contrast, there is no evidence that EHV-9 can cross the immature placenta.

Neurological disease due to EHV-1 infection is often referred to as equine herpesvirus myeloencephalopathy (EHM). The incubation period for EHM is difficult to define because the primary EHV-1 infection might have occurred months before the reactivation event that led to the development of neurological disease [22]. Our observations showed that the neurological signs rapidly progressed within 3–5 days post-inoculation and consisted of tremor, ataxia, and convulsions with paddling as well as nasal discharge, increased and ruffled fur, which indicated that it follows the same pattern of EHV-1 in both duration and clinical signs [23].

Equine herpesvirus-1 is considered a common infectious cause of abortions in horses [22]. Abortions due to EHV-1 infection usually occur in the third trimester. Aborted fetuses do not exhibit evidence of autolysis at the time of abortion since the infected fetuses are viable until shortly before expulsion [24]. The signs of abortion appeared in the late trimester (11th day of pregnancy afterward), even in the early trimester when the fetuses were found dead in uteri (6/36) and (10/26) in early and later trimester infections, respectively, after three days of virus inoculation. At full term, aborted fetuses were dead and appeared congested and undersized with no motility or autolytic changes. Dead fetuses were 4/32 and 12/30 in the early and late trimester infection groups, respectively. Gross lesions on the gravid uterus as well as aborted fetuses at full term were similar to those previously recorded for EHV-9-induced abortions in hamsters and rats [4,5]. The number of dead fetuses in early trimester infections is lower than that observed for late trimester infections, probably due to infections in the late trimester corresponding with a fully developed placenta that facilitates the transmission of infection from dam to fetus.

The histopathological findings in group 2 showed coagulative necrosis of the maternal spaces and stromal decidual cells, especially in the decidua capsularis, at the third dpi in the early trimester infection group. Moreover, dead embryos were degenerated with an ill-developed ectoplacental cone, and the decidua capsularis was severely necrotic with focal dystrophic calcification. Virus antigen was immunohistochemically detected in mononuclear leukocytes in areas of necrotic decidua capsularis. At sixth dpi, dead fetuses were observed. Vacuolation of decidua basalis, vacuolation, necrosis and hemorrhage of spongiotrophoblast layer, and labyrinth with foreign body-type giant cell formation were observed in the placental disc. The mononuclear and necrotic leukocytes in the thin decidua capsularis and basalis were positively stained with EHV-9 antibodies. These findings are compatible with the destruction of immature placentation, and viruses could not cross the placenta. Herpesviruses implicated in pregnancy loss in the early trimester are herpes simplex virus (HSV) [25] and cytomegalovirus (CMV) [26] in humans. The mechanism of EHV-9 infection involved in early pregnancy loss in the early trimester might be due to the triggering of chemical mediators such as IFN-γ. IFN-γ inhibited the outgrowth of trophoblasts and induced degeneration of implanted blastocysts [27]. EHV-9 might be associated with increased natural killer cell activity, which has been implicated in pregnancy loss [28]. Herpes virus infection is associated with necrosis and enhanced apoptosis in decidual and trophoblastic tissues, which is suggested to be a possible mechanism of pregnancy loss [29]. At the 11th dpi, multifocal necrosis of chorionic villi and spongiotrophoblast areas associated with mononuclear cells near the junction of the uterine wall and trophospongium was observed. The cervical epithelium was necrotic. None of the dead or live fetuses showed pathological lesions. IHC, focal necrotic chorionic villi, chorionic epithelium, and mononuclear cells in the lumen and around the maternal blood vessels as well as the necrotic epithelium of the cervix were stained positive. Necrosis and apoptosis of the chorionic epithelium as well as detection of virus antigen in these cells is a clue that the virus is responsible for abortion in early trimester infection. The first-trimester chorionic villi and isolated cytotrophoblasts were experimentally inoculated with CMV in vitro. Cytotrophoblasts were infected with CMV both in utero and in vitro [26]. It has been suggested that CMV is transmitted to the fetus through crossing syncytiotrophoblasts with subsequent infection of the underlying cytotrophoblasts and through invasive cytotrophoblasts within the uterine wall [26]. In the present study, EHV-9 was detected in mononuclear cells infiltrating necrotic syncytiotrophoblasts in the decidua capsularis and basal tissues. HSV was *hybridized* in situ in the nuclei of the decidual and intermediate trophoblastic cells and less commonly in chorionic villus trophoblastic cells in first trimester pregnancy loss [25]. In the present study, EHV-9 was detected in the chorionic villous epithelium at the 11th DPI. Similarly, in mice inoculated with EHV-1 at different stages of gestation, dams suffered from respiratory distress, weight loss, and nervous signs [30]. When the virus was inoculated at 15 and 17 days of gestation, the dams aborted dead or dying fetuses and the virus was isolated from their placental and fetal tissues. However, inoculation of the virus at the seventh and ninth day of gestation revealed early fetal death and resorption, and the virus antigen was detected in reabsorbed tissue. In contrast, viruses could not be detected in the few aborted fetuses and placentas in this group [30]. Virus DNA and antigens were [5] detected in both the placentas and fetuses when EHV-9 was inoculated in rats in both the early and late trimesters; however, in the present study, we failed to detect both antigens and DNA in fetuses but not in the placenta and uterus when the virus was inoculated in the early trimester in hamsters. This inconsistency in the result might be due to species differences, dose, or inadequate sampling, as we did not examine all offspring, and PCR was applied for only the 11th dpi point of time. Moreover, [4] we could not detect the virus DNA from either the placentas or fetuses of mice and hamsters inoculated with EHV-9 at the early and late trimesters. EHV-1 was isolated from a few placentas (3/72), fetuses (1/73), and uteruses (1/16) of dam mice inoculated with EHV-1 on day 15 of gestation and sacrificed within 5 DPI [31].

In the late trimester, minimal changes occurred in the placentation cone at the third dpi. Virus antigen was detected in the chorionic epithelium of some mice. At the fifth dpi, degeneration of chorionic villi and trophoblasts in the trophospongium was observed. Virus antigen was detected in chorionic villi, a few trophoblasts, and mononuclear cells around necrotic maternal tissue and in some fetal tissues. Congestion, necrosis of the trophospongium, and chorionic necrosis were recorded in EHV-1 induced abortion in mice inoculated in the last trimester (15 days of gestation) [31]. Focal necrosis of the trophospongium and giant cell layer trophoblasts was previously recorded in EHV-9 inoculated mice, hamsters, rats [4,5], and in mice inoculated with EHV-1 [31]. The lesions in the present study involved mainly the placenta, but fetuses were almost free despite the detection of viral antigens in their lungs and liver. These findings confirm the results of previous studies on EHV-9 induced abortion in murine models [4,5]. In contrast, EHV-1 experimental infection in pregnant mares affected the endometrial blood vessels, inducing thrombotic ischemic lesions [32].

In the present study, the antigen was detected in placental and fetal tissues, but not in the uterine wall. Antigens were not detected in maternal and fetal blood vessels. No thrombo-occlusive lesions were detected in EHV-9-induced abortion in the present study and in mice, hamsters, and rats [4,5]. Abortion in EHV-1 is either due to fetal and/or placental infection or placental infarction due to EHV-1-induced endothelial damage. The thrombo-occlusive lesions in the uterine wall were observed on EHV-1 experimental inoculation in ponies; concurrently, no virus antigen or DNA could be detected or isolated from aborted fetuses and placentas [32,33]. [34] Edington et al. (1991) suggested that EHV-1 infection of maternal endothelial cells plays a major role in the pathogenesis of abortion and that endothelial cells are also involved in the dissemination of virus within the fetus. In contrast, EHV-1 has been isolated from a wide variety of dam and fetal tissues after abortion in natural infection, supporting the direct effect of the virus on the placenta and fetuses. The findings of the present study support the direct damage effect of EHV-9 on placental and fetal tissues in pregnant hamsters. Early studies on the inoculation of EHV-1 into pregnant hamsters revealed that placentas were infected with the virus, but fetuses were free of it. The hamsters had died because of hepatic failure [35]. In contrast, viruses have been detected and isolated from both placentas and aborted fetuses after inoculation of EHV-1 in pregnant mice [36] and in neonatal foals in a perinatal infection with EHV-1 [37] and in dead, stillbirth, and alive foals that died within a few days with bronchiolitis, pneumonitis, and hepatic, adrenal, and splenic microscopic necrosis associated with intranuclear inclusions after prenatal EHV-1 infection outbreak [38].

The inoculation of EHV-9 either in the early or late trimester did not affect the gestation period of the inoculated hamsters. These findings are compatible with those reported in mice inoculated with EHV-1 [39] and in pregnant mares perinatally infected with EHV-1 where abortion occurred [40]. In contrast, the authors of [32] found that mice infected with EHV-1 at days 15–17 of pregnancy gave birth significantly earlier than uninfected control mice. In the present study, EHV-9 antigen and DNA were detected in the placenta and uterus, but not in the early trimester, although lesions in placentas were more severe than those of dams inoculated in the late trimester where both placentas and fetuses were positive for virus antigen and DNA. These findings indicated an inconsistency between the severity of the pathological lesions reported in the placenta and recovery of the virus in fetuses, but they are consistent with the fact that the virus can transmit through the fully developed placenta. These findings are compatible with those recorded in rats and mice [5,39].

Viral antigen and DNA of EHV-9 were detected in the liver and lungs of aborted fetuses from dams inoculated in the last trimester and not in the early trimester. Similarly, the lungs and livers of aborted fetuses obtained from mice dams inoculated with EHV-1 in the last trimester showed cytopathic effects of the virus as well as virus antigen [30]. In contrast, the authors of [4] detected virus antigen in the lungs of fetuses aborted from dam hamsters inoculated at the first trimester; however, virus antigen as well as DNA could not be detected in the placentas and fetuses of dams inoculated at the second trimester. Antigens were detected in the lungs and macrophages of the skin of fetuses obtained from rat dams inoculated in the late trimester [5].

Viremia was detected 5–9 days post-infection with an average of 1–4 days in both early and late trimester infections. Viremia has been recorded similarly 1–4 days after experimental infection of the Ab4p strain of EHV-1 in pregnant mares [41] indicating that both EHV-9 and EHV-1 follow the same pattern of infection. Viral DNA was detected in all brain samples of dams starting from the 3rd dpi throughout the experiment until full birth, indicating that after intranasal inoculation of EHV-9, the virus entered through the nasal mucosa along the olfactory pathway and spread transynaptically via its connections to the hippocampus, amygdala, and cerebral cortex [3]. In the uterine and placental tissues, the viral DNA was detected mainly in the full term (11th dpi, 15th day of pregnancy) in the early trimester infected group; however, the virus was detected earlier and in the full term (at 3 dpi, 13th days after pregnancy) in the late trimester infected group. This may be correlated to late pregnancy immunosuppression, which might be responsible for the induction of abortion in the late trimester [42]. The cell-mediated immune response of mares in the later stages of pregnancy to EHV-1 was suppressed, although antibody titers increased as much as 16-fold following exposure to virulent equine herpesvirus 1 [42]. The cell-mediated immune and antibody responses of horses of varying ages and pregnant horses against equine herpesvirus 1 antigen could not be detected in the mare’s uterus beyond 48 h after abortion of an infected fetus [32].

Furthermore, we detected the viral DNA in maternal lung and liver tissues as they were considered to be the most common predilection sites during secondary viremia. In our study, we detected only the viral DNA in only two fetal liver tissue samples, while it could not be detected in the fetal lung and brain. This is different from previous reports about EHV-1 detection in fetal tissue that could be detected in most fetal tissues such as the brain, lung, liver, and thymus from aborted fetuses after EHV-1 infection. Therefore, we tried to obtain a better understanding of this result using quantitative real-time PCR to measure the viral load in different maternal and fetal tissues.

Cytokines are small glycoproteins produced by some cell types, predominantly leukocytes that regulate immunity, inflammation, and hematopoiesis. They regulate a number of physiological and pathological functions, including innate immunity, acquired immunity, and a plethora of inflammatory responses [43]. IFN-γ modulates some components of the immune response. It promotes the activity of cytolytic T lymphocytes, macrophages, and natural killer (NK) cells. Early host defense against infection is likely to utilize IFN-γ secreted by NK cells. In acquired immune responses, T lymphocytes are the major source of IFN-γ [43]. The IFN-γ-dependent immunoregulatory cytokine response increased mainly after EHV-1 infection, especially in non-vaccinated ponies, as a protective immunity against EHV-1 infection [44]. However, the role of IFN-γ in the abortion caused by EHV-9 has not been fully elucidated. In *Brucella abortus* infection, transient IFN-γ production during the period of placental development might trigger abortion. Significantly increased levels of IFN-γ at the third dpi of EHV-9 in both groups 2 and 3 may be due to humoral responses against infection. In contrast, IFN-γ has a protective role in *Neospora caninum-*induced abortion in cows. IFN-γ production protects against abortion in Neospora-infected cows, although it reduces the humoral immune response against *N. caninum* during gestation [45]. Our results showed a significant increase in IFN-γ levels in group 3 during full-term abortion, which might clarify the role of IFN-γ in EHV-9 abortion [46].

Concerning the IFN-γ result after EHV-9 infection, a significant increase in the level of IFN-γ was observed in the control animals in comparison with their levels in dams at the third dpi in both early and late infected groups, which is considered a normal response to viral infection [44]. In addition, the level of IFN-γ increased significantly in G3, during which the abortion occurred at the late stage of pregnancy, which was associated with the full development of the placenta, implementing the role of IFN-γ in EHV-9 -induced abortion. Moreover, a significant increase of TNF-α at the 11th DPI and 6th DPI of EHV-9 occurred in G2 and G3, respectively, suggesting its role in the induction of abortion for two reasons. First, TNF-α induced vascular endothelial cell express new adhesion molecules. This increased the mobilization and effector function of neutrophils and their adhesiveness to endothelial cells, leading to vasculitis and vascular thrombosis in the placental and uterine tissue. Second, increased levels of TNF-α stimulated the release of corticotrophin-releasing hormone, which itself has a role in the induction of abortion, with an increasing role in the presence of TNF-α [43]. The major cell source of TNF-α is macrophages, specifically endotoxin-activated mononuclear phagocytes. [43]. Our results showed significant increases in the level of TNF-α in the experimental group than in the control groups and previous time points in the different time points detected during gestation in both the early and late infected pregnant groups, with the local increases of concentrations of TNF-α causing heat, swelling, redness, and pain. TNF-α might induce vascular endothelial cell damage to express new adhesion molecules. It increases the mobilization and effector function of neutrophils and their adhesiveness for endothelial cells; therefore, this is an important factor in the vasculitis and blood vessel thrombosis in the placental and uterine tissue. It exerts an IFN-like protective effect against viruses and augments expression of MHC class I molecules. TNF-α is an endogenous pyrogen that acts on cells in hypothalamic regulatory regions of the brain to induce fever. The hypothalamic–pituitary–adrenal axis is stimulated via the release of corticotrophin-releasing hormone by TNF-α [43], and the corticotrophin hormones have a role in the induction of abortion, especially with increased levels of TNF-α.

## 5. Conclusions

In conclusion, the present study revealed that in the early trimester, EHV-9 cannot cross through the capsular placenta but can cross through the developed decidual placentation. Abortion in EHV9 occurred through necrosis of the chorionic villi. In addition, the changes in the pathological changes in fetal tissue as well as the placenta may be related to changes in the expression of inflammatory cytokines. These results provide new insights into the pathogenesis of abortion during EHV-9 infection.

## Figures and Tables

**Table 1:** Experimental design.

Group	Date of Gestation at Time of Inoculation	(Number of Animals)/Date of Sampling	Dam’s Number Referred to in Text
Group 1 (control group)		(3) at 8th day of gestation (3) at 11th day of gestation (3) at 13th day of gestation (3) at 15th day of gestation	
Group 2 (1st trimester group)	5th day of gestation	(3) at 8th day of gestation/(3 day post inoculation dpi) (3) at 11th day of gestation/(6 dpi) (3) at 16th day of gestation/(11 dpi)	1, 2 and 3 4, 5 and 6 7, 8 and 9
Group 3 (3rd trimester group)		(3) at 13th day of gestation/(3 dpi) (3) at 16 day of gestation/(5 dpi)	10, 11 and 12 13, 14 and 15

dpi (day post-inoculation).

**Table 2:** Clinical signs, gross lesions, and the number of dead fetuses in hamster dams inoculated intranasally with EHV-9.

Dam No.	Clinical Signs	Gross Lesions	Number of Dead Fetuses/Total Number of Fetuses	Number of Fetuses in Corresponding Control
1	Depression	Petechial hemorrhages on lungs Pregnancy in one horn	0/4	0/10
2	Nasal discharge, mild nervous manifestation (tilting, tremors of head, etc.)	6 pregnancy cysts in the right horn; 2 appeared as transparent empty vesicles. Left horn had one pregnancy cyst	0/7	0/11
3	Nasal discharge and depression	Congested uterus	0/11	0/10
4	Nasal discharge, convulsions	Petechial hemorrhage on lungs, 3 pale, smaller pregnancy cysts	3/11	0/11
5	Nasal discharge, nervous manifestation	Hepatomegaly, pinpoint necrotic foci on the surface of the liver, 2 pregnancy cysts were pale and smaller in size than others.	2/11	0/13
6	Nasal discharge, paddling of the hind limb	Ascites and congested uterus	1/14	0/10
7	Ruffled hair, nasal discharge, nervous manifestation	Congested uterus with dark red areas. 3 undersized dark congested dead fetuses. Petechial hemorrhage on lungs.	3/10	0/8
8	Nervous manifestation	One dead undersized dark fetus	1/11	0/10
9	Nervous manifestation	Pinpoint pale areas on the lungs. No gross changes on uterus or fetuses	0/11	0/10
10	Nasal discharges, convulsions, and tremors	The liver was congested with sharp demarcation of hepatic lobules, pregnancy in one horn	3/6	0/6
11	Ruffled fur, nasal discharge, and convulsions	Congested blackish uterine wall	3/10	0/10
12	Nervous manifestation with hind limb paralysis	Hepatomegaly with hepatic congestion	4/10	0/11
13	Convulsions	Congested liver and brain. Dark red uterus contained 4 undersized dead dark fetuses	4/11	0/8
14	Nervous manifestation and recumbency	Ascites and congested enlarged liver	5/10	0/10
15	Respiratory distress and nervous manifestations	Dark red uterus contained 3 undersized fetuses	3/9	0/10

**Table 3:** Summary of the immunohistochemical detection of EHV-9 antigen and PCR amplification of EHV-9 ORF30 gene from different organs, fetuses, placentas, and uterine walls of pregnant hamster dams intranasally-inoculated with EHV-9.

Dam No.	IHC					PCR						
	Brain	Lungs	Liver	Fetus ^a^	Placent ^a^	Blood	Brain	Lungs	Liver	Uterus ^b^	Placenta	Fetus
1	+	−	−	−	−	−	+	−	−	−	NA	NA
2	+	−	−	−	−	−	+	−	−	−	NA	NA
3	+	−/+	−	−	+	+	+	−	−	−	NA	NA
4	+	+	−	−	−/+	+	+	+	−	−	NA	NA
5	+	+	+	−	+	+	+	+	+	+	NA	NA
6	+	−	−	−	−/+	+	+	−	−	−	NA	NA
7	+	+	+	−	++	−	+	+	+	+	+	−
8	+	−	+	−	++	−	+	−	+	+	+	−
9	+	+	−	−	+	−	+	+	−	+	+	−
10	+	−	+/−	−	+	+	+	−	−	+	+	−
11	+	−	−	−	−	−	+	−	−	−	−	−
12	++	+/−	+	H/+	+	+	+	−	+	+	−	−
13	+	+	+	H/+ P/+	++	+	+	+	+	+	+	P/+ H/+
14	+	+	+	H/+ P/+	++	+	+	+	+	+	+	P/+ H/+
15	+/−	−	+	Ep/+ H/+ P/+ In/+	+	+	+	−	+	+	+	−

^a^ Organs in fetuses were positive; ^b^ Whole pregnancy cyst for dams (1–6)/uterine wall for dams (7–15)., + Positive ++ high positive frequency of immunostaining, +/− borderline few cells were stained positive and negative. NA; not applicable. H, P, Ep and In. for hepatic, pulmonary, ependymal, and intestinal tissues, respectively.
